# Pepsin concentration in oral lavage fluid of rabbit reflux model constructed by dilating the lower esophageal sphincter

**DOI:** 10.1515/med-2023-0787

**Published:** 2023-09-21

**Authors:** Zhezhe Sun, Wei Wu, Gang Wang, Lianyong Li, Lei Wang, Hongdan Liu

**Affiliations:** Otolaryngology Head and Neck Surgery Department, Strategic Support Force Medical Center, Beijing, 100101, China; Gastroenterology Department, Strategic Support Force Medical Center, Beijing, 100101, China

**Keywords:** laryngopharyngeal reflux, gastroesophageal reflux, animal model, balloon dilation

## Abstract

The aim of this study was to explore the changes in pH and pepsin concentrations in oral lavage fluid of rabbit reflux model. A total of 18 New Zealand rabbits were randomly divided into two groups. The lower esophageal sphincters (LESs) of the rabbits in the experimental group (EG) were dilated by balloon after the LESs were localized by manometry. The pH levels of the throat and the lower esophagus were monitored 1 week before and 2 weeks after inflation. Oral lavage fluid was collected 1 week before, and 2 and 8 weeks after inflation. The pH monitoring showed that the percentage of reflux time, the number of reflux events, and the longest time of reflux after the dilation (AE) in the EG were significantly higher than before the dilation (*P* < 0.01). The pepsin concentrations at 2 and 8 weeks AE in the EG were significantly higher than that before and that in the control group (*P* < 0.05). Based on receiver operating characteristic curve analysis, the best diagnostic threshold value was 30.3 ng/ml. The reflux model constructed by balloon inflation of the LES in rabbits is characterized by a decrease in throat pH and an increase in salivary pepsin concentration.

## 1 Introduction

Laryngopharyngeal reflux disease (LPRD) refers to the reflux of gastric contents to the part above the upper esophageal sphincter, causing a series of unspecific symptoms such as dry throat, a sensation of something being stuck in the throat, persistent throat clearing and hoarseness, and signs such as subglottic edema, vocal fold edema, and posterior commissure hypertrophy [1]. LPRD is a common disease in the otorhinolaryngology department. Reports from abroad have shown that about 10% of the patients in the otolaryngology clinic have symptoms or signs of laryngopharyngeal reflux [2]. The recent screening of LPRD patients in ENT clinic in China has shown that the incidence rate of LPRD is similar to the aforementioned data [2]. The pathogenesis of LPRD is related to many factors such as anti-reflux barrier, esophageal clearance ability, and sensitivity of target organs [3]. A previous study showed that LPRD could be induced by destroying the anti-reflux barrier structurally and functionally, especially the lower esophageal sphincter (LES), thus resulting in gastric reflux contents back to the upper airway [4,5].

LPRD has received increasing attention among researchers over recent years. In order to further explore the pathophysiological mechanism of this disease, the appropriate animal model is of utmost importance. In the past, some experimental animal models were established [3]. Animal models of LPRD were mostly borrowed from gastroesophageal reflux disease (GERD) animal models, and the establishment methods were mainly endogenous or exogenous [4–7]. An exogenous method is mainly used to construct the model by spraying or injecting one or more substances in gastric acid, pepsin, and bile salt into the throat of experimental animals. This method is simple and feasible with a high survival rate of animals, but it does not conform to the pathophysiology of reflux and cannot fully reveal the occurrence and development of LPRD. Endogenous methods are mainly used to alter gastrointestinal anatomy or break the reflux barrier by surgical methods, causing reflux of gastrointestinal contents. Currently, the most commonly used modeling methods include LES incision or resection [4], pylorus or duodenal constriction [8]. The animal model constructed by this method is more in line with the pathophysiology of reflux, but the operation is difficult, the trauma to animals is greater, and the survival rate of animals is lower. Over recent years, studies have shown that the balloon dilation of LES can be used to safely and effectively construct animal models of GERD and was expected to be used in LPRD research [9].

In this study, the New Zealand rabbit reflux model was constructed by balloon dilation of LES so as to explore the changes of the pH in the throat and pepsin concentration in oral lavage fluid after the dilation (AE).

## 2 Methods

### 2.1 Animals

A total of 18 5-month-old New Zealand white rabbits (purchased from Keyu Animal Breeding Center, Beijing) weighing 2.5–3.0 kg were randomly divided into two groups according to the random number table, including 10 in the experimental group (EG) and 8 in the control group (CG). All rabbits were raised in the appropriate cages at 23 ± 2℃ under a 12-h:12-h light–dark cycle with free access to food and water. The experiment started after the animals adapted to the environment for 3 days. All experimental animals were treated according to the Guide for the Care and Use of Laboratory Animals. This study was approved by the Ethics Committee of the Characteristic Medical Center of the Strategic Support Force of PLA Z2022 Ethical review No. 01.

### 2.2 LES manometry and positioning

Before the inflation, LES manometry and positioning were made with a single lead solid-state esophageal manometry catheter (MMS corporate, the Netherlands). The animals were fasting for 24 h and were denied water for 6 h before the operation. After the animals were anesthetized and fixed, the instrument was calibrated. The manometry catheter was put through the mouth, first letting the electrode down to the stomach. At that time, the pressure was about 10 mmHg, and the pressure curve was straight. The electrode was slightly lifted up until the pressure regularly rose. A mark was made on the wire at the gate teeth at that time and was recorded as the lower edge of LES. An electrode was continued to be slowly lifted up so as to let the pressure to continue to rise, keeping three contraction cycles at each position until the pressure began to drop and was below the intragastric pressure, and regular respiratory waves appeared on the pressure curve. Another mark was made and recorded as the upper edge of LES. The average rest pressure of LES was calculated with the software of the instrument, and the distance between LES and the gate teeth was accurately calculated based on the marks on the wire.

### 2.3 Dilation of LES

The rabbits were fasted for 24 h and were denied water for 6 h before the operation, after which they were anesthetized with ketamine (100 mg/kg i.p.) plus xylazine (10 mg/kg i.p.) and fixed on the operating table. Hercules® Balloon Inflation Catheter (Wilson-Cook Medical Inc. USA, with the largest dilated diameter of 1.5 cm, the effective dilated length of 5.5 cm) was placed through the mouth. The distance between the LES and the incisor was determined by esophageal manometry. The balloon was inserted into the esophagus along the guidewire so that the center of the balloon was at the target position. Water was slowly injected into the balloon such that the process of water injection was not less than 30 s. The final pressure was maintained at 1 atm for 5 min. The dilation was repeated two times with an interval of 3 min. It took about 15 min to dilate. The same procedures were performed in the CG; the balloon was inserted into the esophagus for 15 min but not inflated. During the dilation, ECG and blood oxygen saturation of the animals were monitored. If the heart rate and blood oxygen abnormally decreased, the operation was immediately stopped. The animals fasted for 24 h after the operation, and there was no limit to water intake.

### 2.4 pH monitoring in the throat

pH monitoring was carried 1 week before and 2 weeks after balloon inflation to observe laryngopharyngeal reflux. After the animals were anesthetized and fixed, two Restech DX-pH electrodes (Respiratory Technology Corporation, USA) were placed in the throat through the mouth, and the pharyngeal electrode was put in the hypopharynx. The monitoring time was 2 h.

### 2.5 Determination of salivary pepsin

#### 2.5.1 Retention and preservation of oral lavage fluid

The oral lavage fluid of the experimental rabbits was collected 1 week before the dilation (BE) and 2 and 8 weeks AE. After being anesthetized, the animal was fixed on the operating table. Its head was turned to the right 90°, after which 0.5 ml of physiological saline was taken in a 1 ml sterile syringe and slowly injected into the animal’s mouth and pharynx from the left corner of the mouth. After 10 s, a sterile syringe was used to draw the oral lavage fluid from the right corner of the mouth, infused into a disposable blood collection tube, and stored at −80℃.

#### 2.5.2 ELISA determination of pepsin in rabbit oral lavage fluid

The operation was carried out according to the instructions of the rabbit pepsin ELISA kit (Fu-T227, Beijing Equation Biotechnology Co., Ltd). After thawing, the samples were centrifuged at 3,000 r/min for 10 min. A volume of 10 μl of the supernatant was successively placed in the sample well pre-coated with pepsin antibody, and 40 μl of the sample diluent was added. Standard substance (S0–S5) was at 0, 3, 6, 12, 24, 48 g/l. Then, the antibody and substrate were added in turn. Within 5 min after the reaction’s termination, the optical density (OD) of each well was measured in sequence at the wavelength of 450 nm with an enzyme reader. The standard linear regression curve was drawn on the logarithmic coordinate paper according to the sample OD value, and the corresponding concentration was found out from the standard curve based on the OD value of the sample solution.

### 2.6 Statistical analysis

SPSS 25.0 (IBM, USA) was used for data analysis. For the measurement data that conformed to the normal distribution, the mean ± standard deviation was used, and [M (Q2, Q3)] was used for the non-normal distribution measurement data. For the comparison of normal distribution measurement data between the two groups, the homogeneity test of variance was carried out first. If the variance was uniform, the *t*-test was used; if the variance was not uniform, the *t*′-test was used. For the comparison of normal distribution measurement data among multiple groups, the analysis of variance was used, and for the comparison of non-normal distribution data among multiple groups, the Wilcoxon rank-sum test was used. Chi-square test was used for the counting indexes and binary classification indexes, and the difference was considered statistically significant if *P* < 0.05.

## 3 Results

### 3.1 Data of the experimental animals

In the EG, there were initially ten rabbits, out of which nine survived and one died of pulmonary infection. In the CG, there were initially eight rabbits, out of which seven survived and one died of diarrhea. There was no significant difference in mortality between the two groups (*P* = 0.867). After expansion, the experimental animals showed decreased appetite, reduced activity, and irritability, and some of them salivated. The CG returned to normal after 2–3 days, while the EG returned to normal after 5–7 days. The weight of the EG before and at 2 and 8 weeks after the expansion was 2641.3 ± 65.9 g, 2988.1 ± 81.4 g, and 4074.3 ± 71.0 g, respectively. The body weight of the CG before and at 2 and 8 weeks after the expansion was 2607.1 ± 75.6 g, 3181.8 ± 62.6 g, and 4127.1 ± 29.9 g, respectively. Weight before the expansion was similar between the two groups (*P* = 0.367). Two weeks after the expansion, there was a statistically significant difference in lightweight before the EG and CG (*P* = 0.000). After eating, basic activity returned to normal in two groups of animals, and the weight was gradually restored in the EG at 8 weeks after expansion. The experimental animal body weight was slightly lower than the CG, but there was no statistically significant difference (*P* = 0.091).

### 3.2 Manometry and positioning results of LES

Of the nine rabbits in the EG, the middle point of LES was 24.2 ± 0.8 cm from the incisor, the length of LES was 0.7 ± 0.1 cm, and the average resting pressure of LES BE was 28.0 ± 5.2 mmHg. Of the seven rabbits in the CG, the middle point of LES was 24.1 ± 0.6 cm from the incisor, the length of LES was 0.8 ± 0.2 cm, and the average resting pressure of LES BE was 27.6 ± 3.8 mmHg.

### 3.3 Throat pH monitoring results

**Figure 1:**
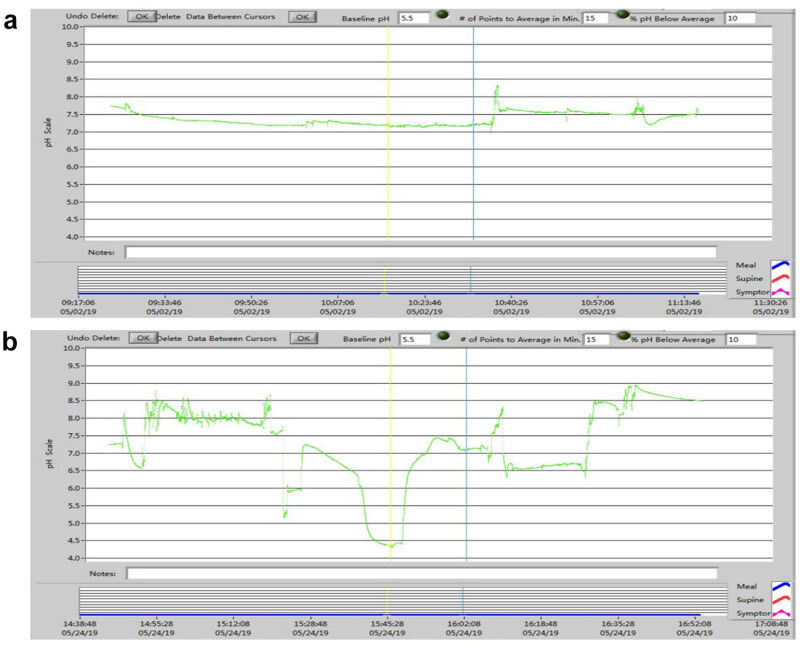
pH monitoring data BE and AE of animal 2 in the EG. (a) No reflux event was found in throat pH monitoring 1 week BE in EG dilation. (b) Acid reflux event with pH < 5 was found in throat pH monitoring 2 weeks AE in EG.

The pH monitoring in the throat BE and AE of LES in the EG showed (Figure 1) that the percentage of acid reflux time with pH < 5, the number of reflux events, and the longest time of reflux events at 2 weeks AE were significantly higher than those BE (*P* < 0.001 in all comparisons) (Table 1). In the CG, there was no statistical significance in the difference of monitoring data before and after sham dilation (*P* > 0.05 in all comparisons). After the dilation, all reflux indexes in the EG were higher than those in the CG, and the differences were statistically significant (*P* < 0.01 in all comparisons) (Table 2).

**Table 1:** Comparison of throat pH monitoring data AE and BE in the EG (*n* = 9)

	1 week BE	2 weeks AE	*Z* value	*P* value
Acid reflux time (%)	0(0,0)	17.5(8.2,29.4)	−3.265	<0.001
Reflux events (times)	0(0,0)	3(1,5.5)	−3.192	<0.001
Longest time of reflux (min)	0(0,0)	17.2(10.2,30.8)	−3.747	<0.001

Note: BE stands for before dilation; AE stands for after dilation.

**Table 2:** Comparison of throat pH monitoring data between EG and CG after modeling

	EG(*n* = 9)	CG(*n* = 7)	*Z* value	*P* value
Acid reflux time (%)	17.5(8.2,29.4)	0(0,3.1)	−2.942	<0.001
Reflux events (times)	3(1,5.5)	0(0,0.5)	−2.598	0.001

Note: EG stands for experimental group; CG stands for control group.

### 3.4 Salivary pepsin assay results

**Figure 2:**
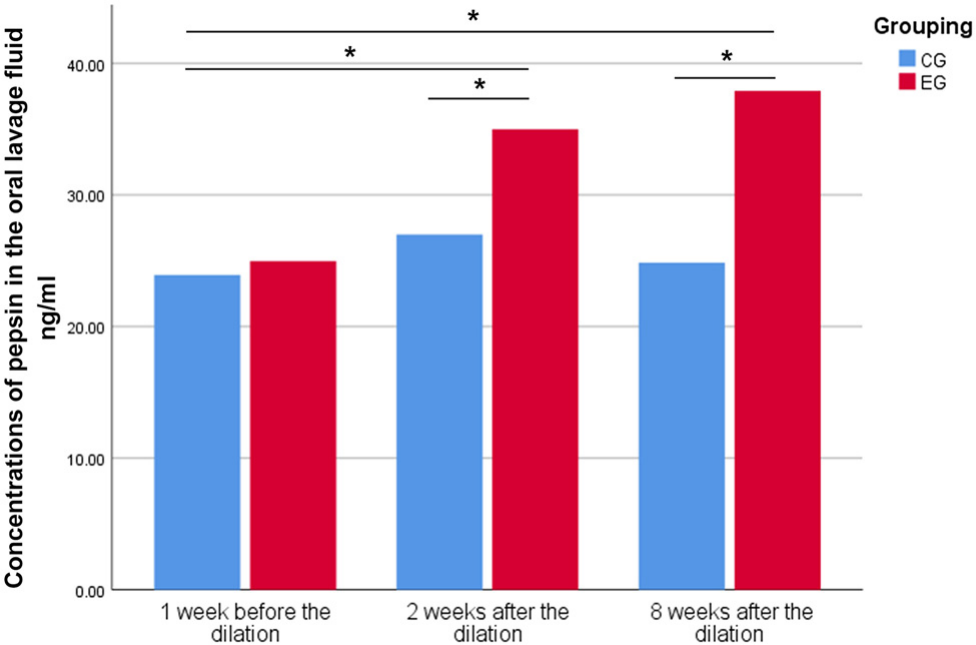
Pepsin concentrations in oral lavage fluid 1 week BE, and at 2 and 8 weeks AE in the experimental animals. *P < 0.05.

There was no significant difference in the concentrations of pepsin among the three-time points in the CG (*P* > 0.05). In the EG, the concentrations of pepsin 2 and 8 weeks AE were higher than BE (all *P* < 0.001), and there was no significant difference between that 2 and 8 weeks AE (*P* = 0.17). There was no significant difference in the concentration of pepsin BE between the EG and CG (*P* = 0.57). The concentrations of pepsin at 2 and 8 weeks AE in the EG were higher than those in the CG (*P* < 0.05) (Figure 2).

**Figure 3:**
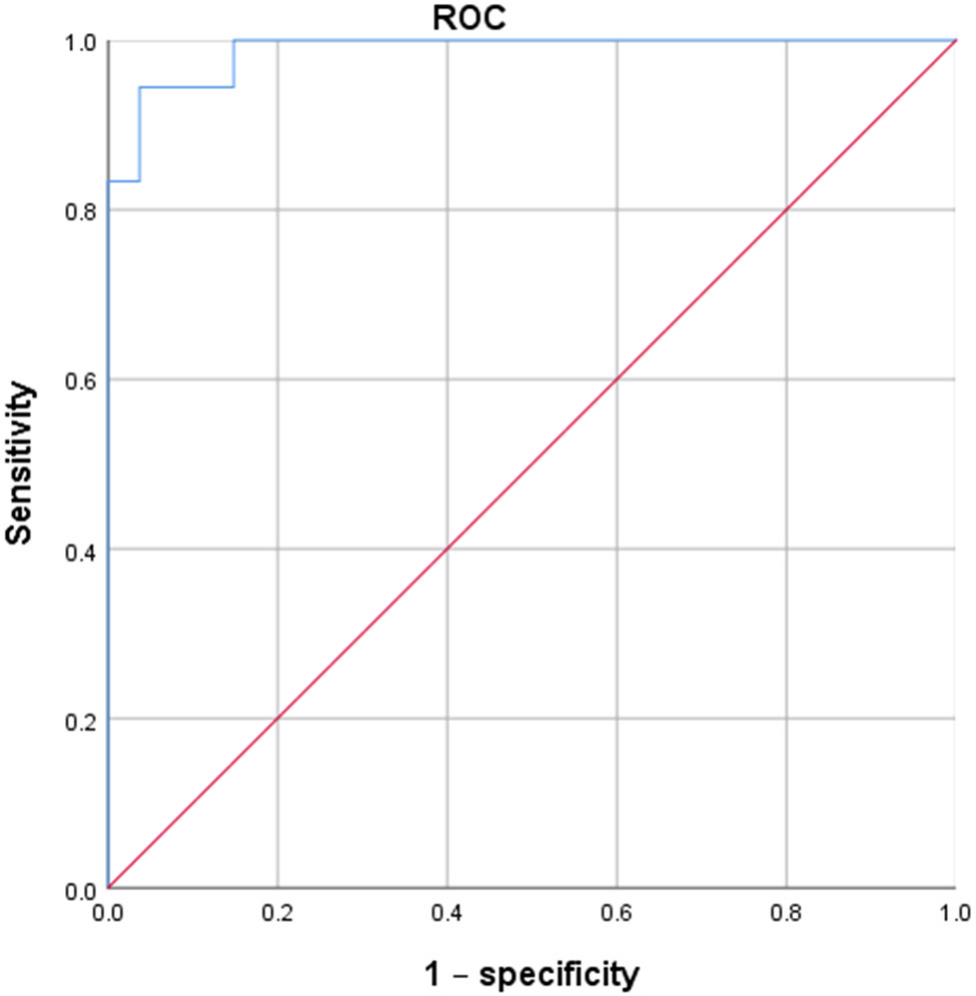
ROC for diagnosis of LPRD based on pepsin concentration in rabbit oral lavage fluid.

Given the significant statistical difference in the pepsin concentration in oral lavage fluid between the EG and the CG, as well as between those BE and AE in the EG, we regarded the pepsin concentration data of the CG at each time point and the BE data in the EG as LPRD negative, and the data at 2 and 8 weeks AE in the EG as LPRD positive so as to make the receiver operating characteristic (ROC) curve (Figure 3) for diagnosis of LPRD based on the pepsin concentration of oral lavage fluid in the rabbits. Our study showed that the area under the curve was 0.988 (95% CI: 0.965–1.000, *P* < 0.001) and the optimal diagnostic threshold was 30.3 ng/ml, with a diagnostic sensitivity of 94.4%, a diagnostic specificity of 96.3%, and a Youden index of 0.907.

## 4 Discussion

LPRD is a common disease in otolaryngology and has been a focus of interest over recent years. The selection and establishment of an effective animal model are very important for the pathophysiological study of the disease, and the endogenous animal model is more consistent with the pathophysiology of reflux. However, the relevant studies are still not complete due to the great difficulty in surgical operation [10]. In this study, we used the LES dilation method to build an animal model of reflux. Animal model of GERD is commonly built by destroying the barrier function of LES [9,11], in which some LES sphincter fibers are elongated or even broken by balloon dilation, resulting in LES relaxation. In physiological conditions, since the intragastric pressure is higher than the esophageal pressure, LES relaxation leads to gastroesophageal reflux, which might lead to LPRD. The difficulty of this method is the accurate positioning and moderate dilation of LES. In the past, most studies fixed the balloon’s position with a rubber band by opening surgery to avoid the looseness and displacement of the balloon [12], which increased the difficulty of modeling and animal mortality. In this study, the method was improved. First, esophageal manometry was used to accurately locate each experimental animal’s LES position, thus avoiding insufficient inflation or esophageal rupture due to inaccurate positioning. Second, we selected a special digestive tract inflation balloon with an effective inflation length of 5.5 cm. The inflated balloon in the effective inflation length was columnar, not easy to shift.

The feasibility of this method was verified by a preliminary experiment before the formal experiment. We found that the inflated balloon’s position was accurate and inflated LES’s whole length without obvious displacement from the pre-experimental X-ray fluoroscopy and open abdominal observation. After being dilated, the lower esophagus becomes translucent, relaxed, or loses tension because the dilation has broken or destroyed some smooth muscles. Hu et al. reported that simply compromising rabbit LES function could induce GERD and LPRD [13] because LES is the most critical component of the reflux barrier. Previous studies have shown that LES transient relaxation is an important cause of GERD and important pathogenesis of LPRD [14]. After LES relaxation, gas or gas–liquid mixed reflux reaches the esophagus, which can cause the rapid dilation of the esophageal wall, causing upper esophageal sphincter relaxation reflection to facilitate the reflux to the throat and cause LPRD [15,16]. This study showed that reflux indexes based on throat pH monitoring of experimental animals after model construction significantly increased compared with that before modeling. It was found that the concentration of pepsin in oral lavage fluid AE in the EG was significantly higher than that in the CG, and the concentration remained at a high level 2 and 8 weeks AE. There was no statistically significant difference between the two time points, which indicated that there was laryngopharyngeal reflux in the experimental animals that remained stable for a long time after the model construction.

Currently, the most commonly used diagnostic methods include reflux symptom scale, reflux sign score, esophageal dual-probe pH monitoring, oropharyngeal pH monitoring, and salivary pepsin monitoring. As pepsin has an essential role in the pathological process of LPRD, more and more scholars use salivary pepsin monitoring to diagnose LPRD [17]. Pepsin is a digestive enzyme secreted by the main cells of the stomach. It is secreted in the form of pepsinogen and activated to pepsin under the action of gastric acid and the activated pepsin. The pepsin activity is related to the pH value of the environment, with the highest enzyme activity at pH 2.0, inactive at pH 6.5, and partially active at pH 2.0–pH 6.5. When the environment’s pH drops from a higher level to below 2.0, 68–90% of activity can be recovered [18]. Pepsin enters the cells through endocytosis and is stored in the vesicles after it reaches the throat. When the environmental pH decreases due to the recurrent laryngopharyngeal reflux, pepsin can reactivate to cause cell damage. Pepsin can damage the reflux barrier of epithelium by consuming carbonic anhydrase III, reducing the expression of heat shock protein and squamous epithelial protein Sep70, destroying cell connections, and so on [19]. Pepsin that has entered cells can also promote excessive reactive oxygen species and other inflammatory factors to destroy cell DNA and mitochondria, leading to cell death. Studies have shown that gastric acid and pepsin’s synergistic effect aggravates the damage caused by gastroesophageal reflux on the throat mucosa [20]. Pepsin is a specific marker of gastroesophageal reflux that has an important role in the pathological process of LPRD; however, studies on salivary pepsin in animal reflux models have been rarely reported in the past. Due to the small amount of saliva in rabbits, this study used a fixed amount of normal saline to lavage rabbits’ oral cavity and get oral lavage fluid. Our results showed that there was a significant difference in salivary pepsin concentration BE and AE between the EG and the CG. ROC analysis showed that the diagnostic sensitivity was 94.4% and the diagnostic specificity was 96.3% when 30.0 ng/ml was used as diagnostic threshold. Furthermore, salivary pepsin was a reliable index to judge whether the model was successful or not in the animal experiments with high controllable experimental conditions.

In conclusion, the animal reflux model constructed by balloon dilation can be used to confirm the existence of LPRD based on pH monitoring and pepsin measurement in oral lavage fluid. The concentration of pepsin in oral lavage fluid obtained by quantitative saline lavage was stable and credible, thus could be used for further study of LPRD.
